# Exopolysaccharide β-(2,6)-levan-type fructans have a molecular-weight-dependent modulatory effect on Toll-like receptor signalling†

**DOI:** 10.1039/d3fo03066k

**Published:** 2023-12-18

**Authors:** Renate Akkerman, Marjolein M. P. Oerlemans, Michela Ferrari, Cynthia Fernández-Lainez, Bart J. de Haan, Marijke M. Faas, Marthe T. C. Walvoort, Paul de Vos

**Affiliations:** a Department of Pathology and Medical Biology, University Medical Center Groningen Groningen The Netherlands r.akkerman@umcg.nl; b Department of Chemical Biology, Stratingh Institute for Chemistry, University of Groningen Groningen The Netherlands; c Laboratorio de Errores Innatos del Metabolismo y Tamiz, Instituto Nacional de Pediatría Ciudad de México Mexico; d Posgrado en Ciencias Biológicas, Universidad Nacional Autónoma de México UNAM Ciudad de México Mexico

## Abstract

Scope: Fructans are a group of dietary fibers which are known to have many beneficial effects including immune-modulating effects. A family of fructans are β-(2,6)-linked levan-type fructans that are known to serve as exopolysaccharides in the cell wall of many species of bacteria including commensal bacteria and probiotics. It is still largely unknown whether and how they can serve as immunomodulating molecules. Results: Microbial β-(2,6)-fructans were found to induce TLR-dependent activation of THP-1 cells, in a dose-dependent fashion. Low molecular weight (*M*_w_), medium *M*_w_ and high *M*_w_ β-(2,6)-fructans activated both TLR2 and 4 in a dose- and molecular weight-dependent fashion. In addition, it was found that β-(2,6)-fructans were able to inhibit signalling of various TLRs with the strongest effect on TLR5 and 8, which were inhibited by all the β-(2,6)-fructans in a dose- and molecular weight-dependent fashion. The final effect of this activation and inhibition of TLRs on cytokine responses in human dendritic cells (DCs) was minor which may be explained by the counter-activating effects of the different β-(2,6)-linked levan-type fructans on inhibition of TLR signalling in the DCs. Conclusion: A mechanism by which exopolysaccharide levan β-(2,6)-fructans can be immune-modulating is by impacting TLR signalling. This knowledge could lead to food in which exopolysaccharide levan β-(2,6)-fructans are added for preventing disorders where TLR-signalling is modulated.

## Abstract 2

Fructans are a group of dietary fibers which are known to have many beneficial effects including immune-modulating effects.

## Graphical abstract

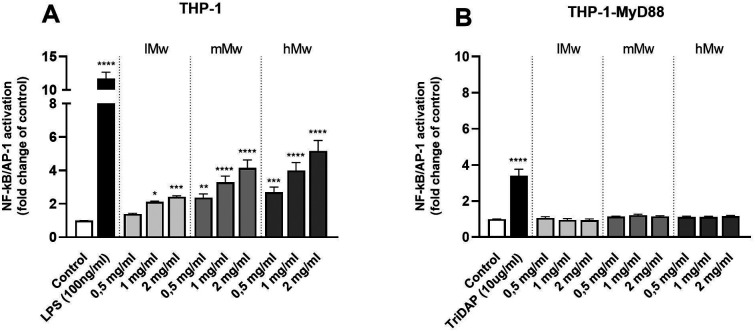

## 1. Introduction

Dietary fibers are complex carbohydrates that resist digestion in the upper intestinal tract. During recent years, many beneficial effects for human health have been attributed to enhanced consumption of dietary fibers. For instance, it has been shown that the intake of a fiber-rich diet contributes to better health and lower mortality in individuals suffering from various inflammatory diseases.^1–3^ Dietary fibers exert their beneficial effect *via* different routes. One of these is through the stimulation of the growth of beneficial intestinal microbes.^4,5^ These beneficial microbes can produce fermentation products like short-chain fatty acids (SCFAs) that can positively influence the mucosal immune system and gut barrier function.^6^ In addition to the microbiota-dependent effects, complex carbohydrates can also directly interact with the intestinal epithelium and immune cells by interacting with their immune receptors such as pattern recognition receptors (PRRs) including Toll-like receptors (TLRs),^5,7–9^ resulting in immune modulation and thereby influencing intestinal immune responses.

Fructans are a group of complex carbohydrates that are often used as a food supplement to serve as a source of dietary fiber.^10^ They are made up of d-fructose residues which can be connected *via* β-(2,1)- or β-(2,6)-linkages to form mostly linear structures.^11^ Depending on the degree of polymerization, fructans can vary considerably in chain length and molecular weight.^11^ Fructans with β-(2,1)-linkages are called inulin-type fructans and are found in many plants.^11^ Fructans with β-(2,6)-linkages are called levan-type fructans and can be found in plants and microbes. Another group of fructans are linked by both β(2 → 1) and β(2 → 6) bonds and are referred to as graminans and are predominantly found in cereals.^12–14^ Much research has been dedicated to microbiota-stimulating and immune-modulating properties of fructans. For example, Logtenberg *et al.* showed that inulin-type fructans can stimulate the growth of Bifidobacterium during fermentation with infant microbiota,^15^ while graminans were shown to have a strong impact on the human gut immune barrier.^16–18^

Levan-type fructans are the most abundant type of fructans on earth.^19^ For example, they are produced by a variety of micro-organisms organisms including Gluconobacter albidus, Lactobacillus reuteri, Helicobactoer hepaticus and *Bacillus subtilis.*^11,20^ Microbial β-(2,6)-fructans can be found on the outer surface of the bacteria, either loosely bound or secreted and are characterized as exopolysaccharides.^11,21^ Although less studied than β-(2,1)-inulin-type fructans, microbial β-(2,6)-fructans are also suggested to exert microbiota stimulating and immune-modulating effects.^21^ For example, microbial β-(2,6)-fructans isolated from *B. subtilis* were shown to enhance the production of proinflammatory TNF-α and the chemokine IL-8 in human-derived ovarian carcinoma cells.^22^ Microbial β-(2,6)-fructans isolated from L. reuteri were shown to increase FOXP3+ CD4+ regulatory T-cells in rats,^23^ and microbial β-(2,6)-fructan isolated from *H. hepaticus* increased the IL-10/IL-6 ratio *in vitro* in bone marrow-derived macrophages.^24^

Although much research has been performed on the possible immunological effects of fructans, the mechanisms underlying their immunomodulatory properties are, especially for β-(2,6)-fructans, less well understood. For β-(2,1)-fructans, several studies have shown that they interact with Toll-like receptors (TLRs), a specific family of pattern recognition receptors (PRRs) present on multiple cell types in the intestine, including epithelial cells and dendritic cells.^7^ Interaction of β-(2,1)-fructans with TLRs can influence the immunological state of these cells.^7^ β-(2,1)-fructans were shown to activate multiple TLRs, including TLR2 and TLR4, and the interaction of the fructans with these TLRs was dependent on the molecular weight (*M*_w_) of the fructans.^7^ Long-chain β-(2,1)-fructans induced a higher activation of TLR2, whereas short-chain β-(2,1)-fructans induced a higher activation of TLR4.^7^ Unfortunately, not much is known about TLR interaction by microbial β-(2,6)-fructans, however, Xu *et al.* suggested that β-(2,6)-fructans can also interact with TLR4, as microbial β-(2,6)-fructans isolated from *B. subtilis* induced Th2 responses in mouse macrophage cells *via* TLR4 signalling.^25^ As there is substantial evidence available showing that β-(2,1)-fructans can interact with TLRs, we hypothesized that specific β-(2,6)-fructans could also have distinct effects on TLR signalling.

Therefore, in this study, we investigated whether microbial β-(2,6)-fructans with varying degrees of *M*_w_ can activate or inhibit TLR signalling. To this end, we used reporter cell lines expressing different TLRs and incubated them with β-(2,6)-fructans. Subsequently, the β-(2,6)-fructans were tested for their cytokine-inducing effects on dendritic cells. In addition, we studied the effect of β-(2,6)-fructans on an intestinal epithelial cell line, after which the conditioned medium of these cells was incubated with DC to study whether soluble products from these intestinal cells affect DC responses.

## 2. Materials and methods

### 2.1 β-Fructans

The low *M*_w_ (lMw) β-(2,6)-fructan (P-Levan) was provided by Megazyme (Wicklow, Ireland), and the medium *M*_w_ (mMw) β-(2,6)-fructans and high *M*_w_ (hMw) β-(2,6)-fructans were kindly provided by Frank Jakob (Technical University of Munich, Germany).

### 2.2 Molecular weight determination

The molecular weight of the β-fructans was measured by gel permeation chromatography (GPC). The analysis was performed on an Agilent Technologies 1200 Series using three PSS Suprema columns (100, 1000, 3000 Å, 300 × 8 mm × 10 μm), with the temperature set at 40 °C. The eluate was monitored by a refractive index (RI) detector. The mobile phase was 0.05 M NaNO_3_ at a flow rate of 1 mL min^−1^. The sample concentration was 2 mg mL^−1^ and the injection volume was 10 μL. Ethylene glycol 0.5% was used as internal standard. Calibration was performed using a pullulan series (PSS-pulkit-12) with a molecular weight in the range of 1.03–708 kDa.

### 2.3 HPAEC-PAD analysis

Samples were hydrolyzed using an adapted method based on a previously described procedure.^26^ In brief, samples were treated with concentrated H_2_SO4 (1 h, 30 °C) and then hydrolyzed with 2.8 M H_2_SO4 for 3 h at 100 °C. Samples were diluted 20 times and 10 μL was directly injected for analysis. High-performance anion exchange chromatography (HPAEC) was performed on a Dionex Ultimate 6000 system (Thermo Scientific, Sunnyvale, CA, USA) equipped with a CarboPac PA-1 column (2 mm × 250 mm ID) in combination with a CarboPac PA-1 guard column (2 mm × 50 mm ID) and pulsed amperometric detection (PAD). The system was controlled by Chromeleon 7.2.9 software (Thermo Scientific, Sunnyvale, CA, USA). Elution of monosaccharides was performed at a flow-rate of 0.25 mL min^−1^ with a multi-step-gradient using the following eluents: A: 0.1 M NaOH, B: 1 M NaOAc in 0.1 M NaOH, C: 0.2 M NaOH, and D: MilliQ water. The gradient used was 16% A, 84% D (20 min), 45% A, 5% B, 50% D (5 min), and 60% A, 40% B (15 min). To regenerate the column, it was flushed for 12 min with 100% C by increasing the flow rate in the first 2 min to 0.35 mL min^−1^. Finally, the column was equilibrated for 12 min with 16% A, 84% D by decreasing the flow rate in the first 2 min to 0.25 mL min^−1^.

### 2.4 Determination of carbohydrate and protein content

The carbohydrate content of the samples was estimated by the phenol-sulphuric acid method, using glucose as a standard.^27^ For the spectroscopic measurements, the absorbance was measured at 490 nm on an Agilent 8453 UV-Visible Spectrophotometer using a 10 mm quartz cuvette. The protein content of the samples was determined by the Bradford assay, using the Bio-Rad assay reagents (catalogue number 500-0116); bovine serum albumin (BSA) was used to generate a standard curve.

### 2.5 Culturing of THP-1 and HEK cell lines

To determine the effects of β-fructans on TLR signalling, various THP-1 and HEK reporter cell lines, all purchased from Invivogen (Toulouse, France), were used. These reporter cell lines express Secreted Embryonic Alkaline Phosphatase (SEAP), which is coupled to the nuclear factor κB/Activating protein-1 (NF-κB/AP-1) promotor. Upon activation of the TLRs by a specific agonist, high levels of intracellular NF-κB will lead to the secretion of SEAP which can be quantified.

To assess the TLR dependent effects of β-fructans, two THP-1 acute monocytic leukemia cell lines were cultured; THP1-XBlue™-MD2-CD14, expressing MD2 and CD14 and thus responding to TLR-ligands and the TH1-XBlue™-DefMyD, expressing a truncated, non-functional form of the TLR adaptor Myeloid differentiation primary response gene 88 (MyD88), and therefore unresponsive to TLR activation. Both THP1 cell lines were cultured in RPMI1640 medium (Gibco, Life Technologies, Bleiswijk, The Netherlands), containing 10% heat inactivated FBS (Fetal Bovine Serum, HyClone, Thermo Scientific, Breda, The Netherlands), 2 mM l-glutamine, 1.5 g L^−1^ sodium bicarbonate (Boom B.V. Meppel, The Netherlands), 4.5 g L^−1^ glucose, 10 mM HEPES, 1.0 mM sodium pyruvate, 100 μg mL^−1^ Normocin™ (Invivogen, Toulouse, Fance), and 50 U mL^−1^ and 50 μg mL^−1^ Penicillin/Streptomycin. All additives were purchased from Sigma Aldrich (Zwijndrecht), unless indicated otherwise.

To investigate signalling *via* individual TLRs, 7 human embryonic kidney (HEK) 293 cell lines (HEK-Blue™-hTLRX) were used, each containing an inserted construct of either human TLR2, 3, 4, 5, 7, 8 or 9. The HEK-Blue-hTLR2 cell line, also expresses TLR1 and TLR6. The HEK cells were cultured in DMEM medium (Gibco, Life Technologies, Bleiswijk, The Netherlands), containing 10% heat inactivated FBS, 2 mM l-glutamine, 4.5 g L^−1^ glucose, 50 U mL^−1^ and 50 mg mL^−1^ penicillin/streptomycin and 100 mg mL^−1^ Normocin. All reporter cell lines were cultured for 3 passages before they were maintained in cell medium containing selective antibiotics, as described before.^28^

### 2.6 Reporter cell line stimulation and inhibition assays and Quanti-blue analysis

THP-1 and HEK cells were seeded into a flat-bottom 96-well plate at a cell density following the manufacturer's protocol (ESI Table 1†). To determine the activation of TLR by β-fructans, cells were stimulated for 24 hours (37 °C, 5% CO_2_) with either 0.5, 1 or 2 mg mL^−1^ β-fructan or the relevant ligand as a positive control (ESI Table 1†). Although all samples tested negative for LPS, we still excluded activation of TLR4 due to contamination with LPS by the addition of 100 μg mL^−1^ polymyxin B to all samples apart from the agonist. For inhibitory properties of β-fructans on the TLRs, cells were stimulated for 1 hour with different concentrations of β-fructans, before exposing cells to the appropriate agonist for 24 hours.

After activation or inhibition, 20 μL of supernatant was mixed with 180 μL of Quanti-Blue in a flat bottom 96-well plate. The plate was incubated for 1 hour (37 °C, 5% CO2) before measuring the absorbance (655 nm) using a VersaMax microplate reader (Molecular Devices GmbH, Biberach an der Riss, Germany) to determine SEAP activity, which represents activation of NF-κB/AP-1. Results are displayed as fold-change compared to the negative control or positive control, for TLR activation and inhibition respectively. As all experiments were done in triplicate and repeated at least 5 times, the mean of the positive or negative controls within each separate experiment was set to 1.

### 2.7 Stimulation of dendritic cells with β-fructans and Caco-spent medium

#### 2.7.1 Cell culture and stimulation

Dendritic cells (DCs) generated from umbilical cord blood CD34 + progenitor cells (hematopoietic stem cells) were purchased from MatTek Corporation (Ashland, MA, USA). DCs were thawed and seeded into 96-well plates at a density of 7 × 10^4^ cells per well and cultured under normal conditions (37 °C, 21% O_2_ and 5% CO_2_) for 24 h according to manufacturer's instructions. After 24 h of culturing, cells were attached to the culture plate after which the medium could be replaced.

To investigate direct stimulation of DCs by β-fructans, DCs were incubated with 200 μL per well DC-MM culture medium (Ashland, MA, USA) containing 0.5 mg mL^−1^ of β-fructans for 48 h. This concentration was selected as the lowest concentration that showed interaction with TLRs. After incubation, supernatants were collected and stored at −20 °C until further analysis. All experiments were repeated five times.

To determine whether intestinal epithelial cells produce soluble signals that may skew the response of DCs, we stimulated intestinal epithelial cells with β-fructans and collected the medium as has been described before.^29^ This Caco-spent medium (CSM) was then used to stimulated DCs. In order to obtain this CSM, Caco-2 cells were seeded in a Transwell system (0.33 cm^2^, pore size 0.4 μm; Corning, Kennebunk, USA) at a density of 20 000 cells per well and cultured under normal conditions for 21 days to reach a stable *trans*-epithelial electrical resistance of >400 Ohm cm^2^. Medium was changed every second day. Caco-2 cells were then incubated with 10 mg mL^−1^ β-fructans on the apical site for 24 hours, after which the basolateral medium was collected and stored at −80 °C (ESI Fig. 1†). The Caco-SM was diluted in DC-MM culture medium at a ratio of 1 : 10. DCs were then incubated with 200 μL per well for 48 hours. After incubation, supernatants were collected and stored at −20 °C until further analysis. All experiments were repeated five times.

#### 2.7.2 Assessment of cytokine production

A magnetic Luminex Assay (R&D systems, Bio-Techne, Minneapolis, USA) was used to determine the levels of MCP-1/CLL2, MIP-1α/CCL3, IL-1β, IL-6, TNFα and IL-10 in the DC supernatant. The assay was performed according to manufacturer's protocol. In brief, cytokine standards were resuspended and serial dilutions were prepared. An antibody magnetic bead mix was added into the wells of a 96-well plate. Standards and samples were added and incubated overnight at 4 °C while shaking. After washing the plate three times, detection antibodies were added and incubated for 30 min at RT while shaking. After incubation, the plate was washed again and incubated with streptavidin-PE for 30 min at RT while shaking. Finally, after the plate was washed again, 100 μL of wash buffer was added to each well. Subsequently, the plate was analyzed using a Luminex 200 System. The data obtained were analyzed using the Luminex xPONENT software. Data were transformed to relative values of the control, which was set to 1.

### 2.8 Statistical analysis

All statistical tests were performed using Prism 9.1.0 software (GraphPad, San Diego, CA, USA). Outliers were removed after testing using a ROUT outlier test (*Q* = 1%). Normal distribution was tested with the Kolmogorov–Smirnov test (*p* > 0.05). In case of the TLR data, if normally distributed, statistical significance was determined with a one-way ANOVA with Dunnett's multiple comparisons test. If data was not normally distributed Kruskal–Wallis test was performed with a Dunn's multiple comparisons test.

For the data of the cytokine production of DCs, samples with cytokine levels that were under the detection level of the Luminex kit were interpreted as 1 : 10 of the lower detection level (ESI Table 2†). Again, outliers were removed after testing using a ROUT outlier test (*Q* = 1%).

The data of cytokine production was normally distributed and analyzed using a One-way ANOVA or a mixed-effects model, with the Geisser-Greenhouse correction, and Dunnett's test (to assess differences between the control and the experimental groups) was used *post hoc*. Significance was set at *p* < 0.001***, *p* < 0.01** and *p* < 0.05*, trends were set at *p* < 0.1.

## 3. Results

### 3.1 Characterization of the tested β-(2,6)-fructans

As determined by gel permeation chromatography (GPC) mMw and hMw β-(2,6)-fructans have a molecular weight in the gigadalton (10^9^ Da) range (Table 1, ESI Fig. 2†), while the IMw β-(2,6)-fructan has a significantly smaller size of 12.5 kDa [Megazyme 9013-95-0].

**Table 1:** Structural analysis of the samples used in this study

Sample	*M*_w_ (kDa) distribution^a^	Monosaccharide composition^b^			Carbohydrate content estimation^c^ (%)	Protein content^d^ (%)
		Gal	Glc	Fru		
lMw β-(2,6)-fructan	12.5^e^	−	+	+	68 ± 0.3	0
mMw β-(2,6)-fructan	405.8 × 10^3^ f	−	−	+	86 ± 2	0
hMw β-(2,6)-fructan	1986.2 × 10^3^ f	−	−	+	75 ± 7	0

a *M*_w_ = Molecular weight as determined by AF4-MALLS (Ua-Arak) and by SEC (Megazyme) and confirmed by GPC (ESI Fig. 2†).

b The monosaccharide composition of the β-(2,6) fructans was determined by HPAEC-PAD analysis. Gal = galactose, Glc = glucose, Fru = fructose.

c Carbohydrate content was determined using the Dubois method (Dubois *et al.*, 1956^27^). Values are the average of three measurements, using a glucose standard curve as a reference.

d Values are the average of three measurements.

e *M*_w_ average data is taken from the product sheet [Megazyme 9013-95-0].

f *M*_w_ data is taken from Ua-Arak *et al.*, 2017.

The monosaccharide composition of the fructans was analyzed by HPEAC-PAD. lMw β-(2,6)-fructan contained mostly fructose and, in lower amount, glucose, whereas only fructose was detectable for mMw β-(2,6)-fructan and hMw β-(2,6)-fructan (Table 1).

The carbohydrate content was estimated using the Dubois method^27^ and was 68%, 86% and 75% for lMw β-(2,6)-fructan, mMw β-(2,6)-fructan, and hMw β-(2,6)-fructan respectively (Table 1).

### 3.2 TLR-induced activation of NF-κB/AP-1 in THP-1 cell lines by β-(2,6)-fructans is dependent on the presence of the TLR adapter MyD88

**Fig. 1:**
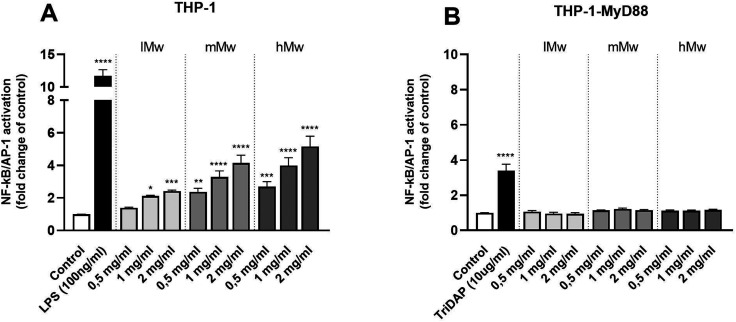
NF-κB/AP-1 activation in THP-1 MD2-CD14 (A) and THP-1 defMyD88 reporter cells (B) by β-(2,6)-fructans. Cells were incubated with 0.5, 1 or 2 mg mL^−1^ β-fructan. Data are expressed as mean ± standard error of the mean (SEM) (*n* = 5). For normally distributed data, statistical significance was determined using a One-way ANOVA test with a Dunnett's multiple comparisons post-test. For not-normally distributed data, a Kruskal–Wallis test followed by a Dunn's multiple comparisons test *post hoc* was done (*P < 0.05, **p < 0.01, ***p < 0.001, ****p < 0.0001).

To investigate the involvement of TLRs in the immune modulatory properties of β-(2,6)-fructans, it was first determined whether the β-(2,6)-fructans can activate THP1-MD2 CD14 cells. These cells express all TLRs. To this end, THP1-MD2 CD14 cells were exposed to 0.5 mg mL^−1^, 1 mg mL^−1^ and 2 mg mL^−1^ of either type of β-(2,6)-fructans. These concentrations were similar to those used in previous TLR studies.^7^ NF-κB/AP-1 expression was determined as a measure for TLR activation (Fig. 1a).

β-(2,6)-Fructans had a strong TLR-activating effect. All concentrations of lMw, mMw, and hMw β-(2,6)-fructans induced NF-κB/AP-1 expression. We observed a 2.4-fold (*p* < 0.1), 4.1-fold (*p* < 0.0001) and 5.1-fold increase (*p* < 0.0001) for the 2 mg mL^−1^ concentrations compared to unstimulated control for lMW, mMw and hMw respectively. The induction of NFκB/AP-1 expression by lMw, mMw and hMw β-(2,6)-fructans was concentration dependent (Fig. 1a).

To determine whether the observed activation of NF-κB/AP-1 was TLR dependent, the β-(2,6)-fructans were also tested on THP-1 myD88-deficient cells. These cells are deficient in MyD88 which is an essential adapter molecule for TLR2, 4, 5, 7, 8, and 9 signalling. None of the β-fructan structures were able to induce a statistically significant activation of these cells illustrating the TLR dependency of NFκB/AP-1 activation (Fig. 1b).

### 3.3 β-(2,6)-Fructans activate TLR2 and 4 in a dose- and molecular weight-dependent fashion

Next, β-fructans were tested for their ability to activate or inhibit specific TLRs. To this end, HEK293 cells expressing either human TLR2, in combination with TLR1 and TLR6, or TLR3, 4, 5, 7, 8, or 9 as single TLR were stimulated with the β-fructans. The NFκB/AP-1 expression was determined and expressed as fold changes compared to the negative control.

**Fig. 2:**
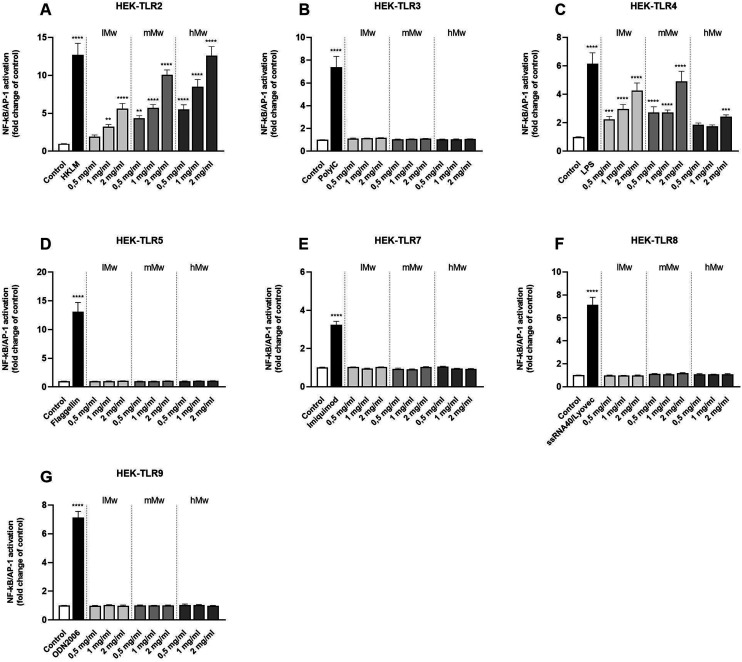
Activation of HEK reporter cell lines expressing individual TLRs (A–G) by β-(2,6)-fructans. HEK cell lines were stimulated with 0.5, 1 and 2 mg mL^−1^ β-fructans. Values are expressed as mean ± standard error of the mean (SEM) (*n* = 5). Statistical significance was determined using a one-way ANOVA with a Dunnet's multiple comparisons test for normally distributed data and a Kruskal–Wallis test with a Dunn's multiple comparisons test for not normally distributed data (**p < 0.01, ***p < 0.001, ****p < 0.0001).

The different β-(2,6)-fructans used in this study (lMw, mMw and hMw) all induced significant activation of TLR2 and 4 in a dose- and molecular weight-dependent fashion (Fig. 2a and c). For TLR2, the lMw, mMw and hMw β-(2,6)-fructans at a concentration of 2 mg mL^−1^ induced a 5.6-fold (*p* < 0.0001), 8.7-fold (*p* < 0.0001) and a 11.1-fold (*p* < 0.0001) increase in receptor activation respectively. For TLR4, the lMw, mMw and hMw β-(2,6)-fructans at a concentration of 2 mg mL^−1^ induced a 4.3-fold (*p* < 0.0001), 4.9-fold (*p* < 0.0001) and a 2.3-fold (*p* < 0.0001) increase in receptor activation respectively. The other TLRs, TLR3, 5, 7, 8 and 9 were not significantly activated by the β-(2,6)-fructans.

### 3.4 β-(2,6)-Fructans inhibit signalling of various TLRs

**Fig. 3:**
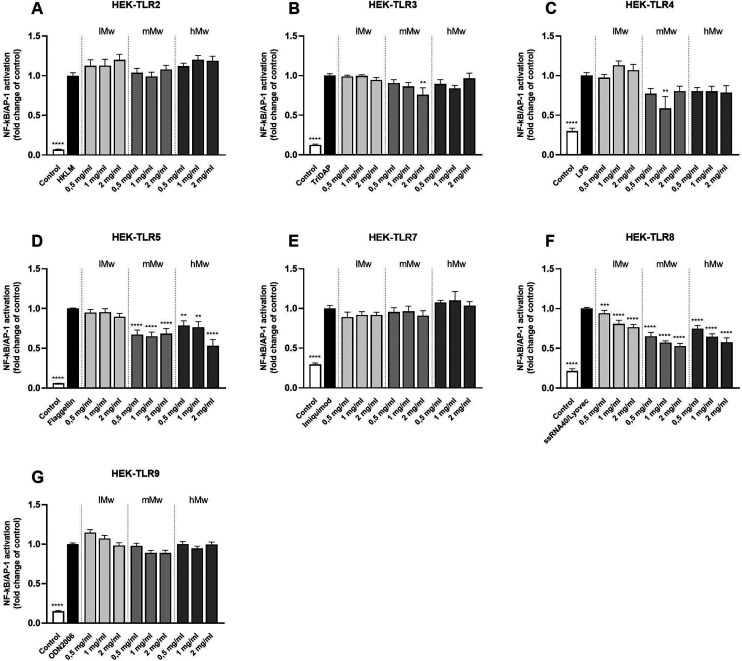
Inhibition of individual TLRs β-(2,6)-fructans. HEK cell lines expressing individual TLRs (A–G) were pre-incubated with 0.5, 1 or 2 mg mL^−1^ β-(2,6)-fructans before stimulation with their agonists. Values are expressed as mean ± standard error of the mean (SEM) (*n* = 3–5). Statistical significance was determined using a one-way ANOVA with a Dunnet's multiple comparisons test for normally distributed data and a Kruskal–Wallis test with a Dunn's multiple comparisons test for not normally distributed data (**p < 0.01, ***p < 0.001, ****p < 0.0001).

To determine the inhibitory properties of β-fructans on TLR signalling, HEK cells were pre-incubated with β-fructans for 1 hour before stimulating them with their respective TLR agonists. Inhibition of β-(2,6)-fructans was quantified by comparing the NF-κB/AP-1 expression in presence of the fructans to the control containing only the agonist (Fig. 3).

β-(2,6)-Fructans showed strong inhibitory properties on different TLRs in a *M*_w_ dependent fashion. mMw β-(2,6)-fructans, at a concentration of 2 mg mL^−1^ inhibited TLR3 to 0.76-fold (*p* < 0.01). The 1 mg mL^−1^ concentration of the mMw β-(2,6)-fructan also inhibited TLR4 to 0.59-fold (*p* < 0.01). Also, the mMW and hMw β-(2,6)-fructans inhibited TLR5. This effect was seen for all concentrations. The 2 mg mL^−1^ concentrations of the mMw and hMw β-(2,6)-fructans inhibited TLR5 up to 0.68-fold (*p* < 0.0001) and 0.53-fold (*p* < 0.0001) respectively. TLR8 was inhibited by all concentrations of the lMw, mMw, hMw β-(2,6)-fructans up to a 0.77-fold (*p* < 0.0001), 0.53-fold (*p* < 0.0001) and 0.58-fold (*p* < 0.0001) respectively.

### 3.5 Stimulation of dendritic cell with β-(2,6)-fructans directly or indirectly and induced cytokine production

**Fig. 4:**
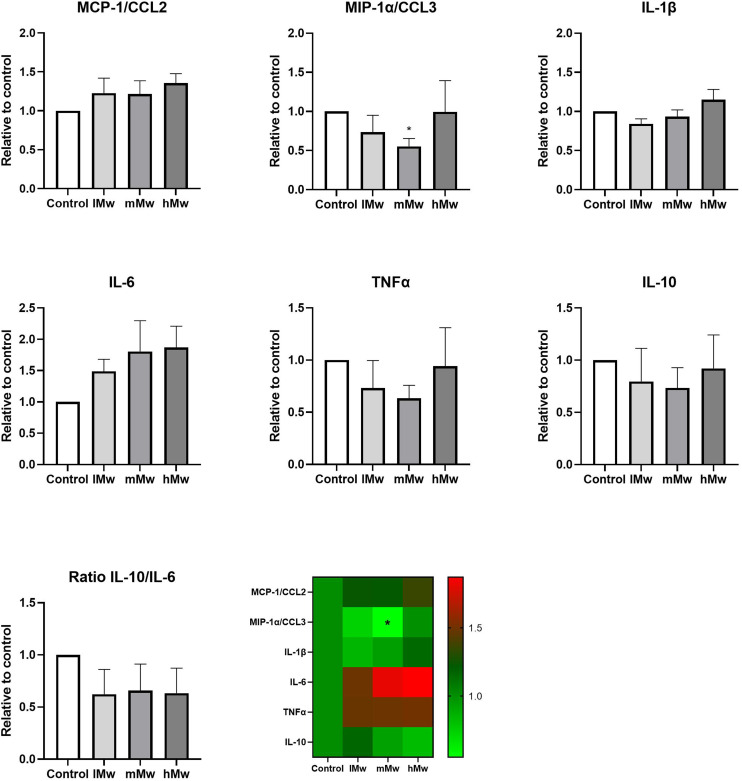
Cytokine production by dendritic cells incubated with β-(2,6)-fructans. Dendritic cells were incubated with 0.5 mg mL^−1^ of β-fructans for 48 h. Subsequently, cytokine levels in the supernatant were determined using Luminex. In addition, the ratio for IL-10/IL-6 was determined. Values are expressed as mean ± standard error of the mean (SEM) (*n* = 5). Statistical significance was determined using a One-way ANOVA test followed by a Dunnett's multiple comparison test.

Dendritic cells are key players in the gut mucosal immune system and are distributed along the intestinal epithelium.^30^ Therefore, we investigated whether the β-(2,6)-fructans could also influence cytokine production of DCs. To this end, we incubated DCs for 48 h in the presence and absence of the β-fructans and determined cytokine release. However, only the production of MIP1α/CCL3 was significantly reduced by the incubation with mMw β-(2,6)-fructans. No further significant differences were observed (Fig. 4).

**Fig. 5:**
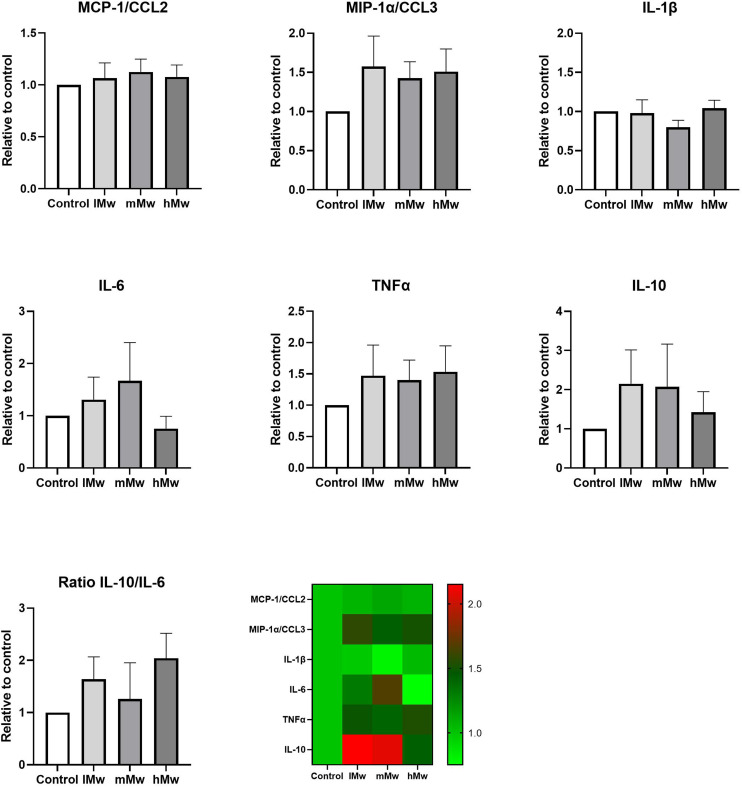
Cytokine production by dendritic cells incubated with Caco-spent medium of β-(2,6)-fructans. Dendritic cells were incubated with of β-(2,6)-fructans CSM. Values are expressed as mean ± standard error of the mean (SEM) (*n* = 5). Statistical significance was determined using a mixed effects analysis followed by a Dunnett's multiple comparison test. No significant differences were found.

To investigate whether the β-fructans could influence DC cytokine production *via* intestinal epithelial cell derived factors, DC were incubated with β-(2,1)- and β-(2,6)-fructans Caco-spentd medium (1 : 10 diluted) for 48 h. These incubations also did not induce significant differences in DC cytokine production (Fig. 5).

## 4. Discussion and conclusion

Fructans are complex carbohydrates that exert many beneficial effects on the human health. β-(2,1)-fructans are extensively investigated for their effect on the immune response and have been shown to interact with TLRs to elicit their response.^21^ Although β-(2,6)-fructans presented similar effects on the immune response in human *in vitro* studies,^22,24,25^ the mechanisms underlying the immunomodulatory function of β-(2,6)-fructans are not well understood. Since it was shown that β-(2,1)-fructans elicit their immunomodulatory response through TLR interaction,^7,29^ we hypothesized that similar mechanisms might be involved in the immunomodulatory effects of β-(2,6)-fructans. Therefore, this study was undertaken to determine the potential immunomodulatory effects of exopolysaccharide β-(2,6)-levan-type fructans and the mechanisms involved.

Levans are the most abundant fructans on earth and are a pertinent component of both plants and microbes that humans consume on a daily basis.^11^ They are also part of commensal bacteria. Among these are Gluconobacter albidus, Lactobacillus reuteri, Helicobactoer hepaticus, *Bacillus subtilis* and Bifidobacterium longum,^11,20^ but also Streptococcus salivarius in the oral cavity,^31^ and Lactobacillus reuteri.^32^ The levans on these organisms can be found either in the cytoplasm or integrated into the cell wall as capsular exopolysaccharide.^33–35^ They can be either attached to other macromolecules in the cell membrane as glycolipid or glycoprotein or be secreted to exert their beneficial effects.^21^ Many functions have been attributed to exopolysaccharides such as levans which include biological functions such as forming a pertinent anchoring site for commensal bacteria and by that contributing to biofilm formation.^36,37^

Levan-type fructans have been recognized for their immunomodulatory effect,^38–41^ but mechanisms are still largely unknown. They have been shown to modulate cytokine production by immune cells *in vitro*^39–42^ which suggests direct interaction of fructans with immune cells. During recent years we and other have shown that pattern recognition receptors such as TLRs are important targets for glycosidic structures such as fructan.^8,43^ Here we show, to the best of our knowledge for the first time, that levan-type β-(2,6)-fructans influence the signalling of specific immune receptors in a molecular weight-dependent and thus structure dependent fashion. We found that levan-type β-(2,6)-fructans have strong stimulating effects for TLR2 and TLR4. More specifically, we observed that the lMw, mMw and hMw β-(2,6)-fructans could stimulate both receptors in a *M*_w_- and concentration-dependent fashion. In addition, we also found that all β-(2,6)-fructans inhibited TLR8 and that mMw and hMW β-(2,6)-fructans inhibited TLR5. As we used specific reporter cell lines, we know these effects are dependent on the NF-κB/AP1 pathway. However, when β-(2,6)-fructans were incubated with DCs, no effect on cytokine secretion was observed.

TLR2 is a rather versatile receptor containing large areas of binding surfaces with many insertions and β-sheets included 19 leucine rich repeats, to which many molecules might bind.^28^ All three molecular weight β-(2,6)-fructans were able to stimulate TLR2 signalling but this was gradually stronger with the higher molecular weight levans. This could be explained by the clustering of different TLR2 receptors that have a stronger impact when combinedly activated than individual receptors. Similar activation patterns of activation with clustering of receptors have been reported for β-glucans that interact with the PRR Dectin-1.^44^ This could possibly be further investigated using 3D-modelling techniques, however, at this moment, both the 3D-structure of the ligand binding-site as well as the 3D-structure of the area around the binding-site are still unknown.

The pattern of activation of TLR4 was different from that of TLR2. The two lower molecular weights β-(2,6)-fructans gradually increased activation in a concentration-dependent fashion while it was lower at higher molecular weights. This might be related to the pertinent differences in structure between TLR2 and TLR4. TLR4 contains 22 leucine-rich repeats that are suggested to be responsible for the specificity of ligand binding.^45^ An important difference with TLR2 is however that the β-sheet of the central domain has smaller radii and larger twist angles.^46^ Our data suggest that longer molecules might be less readily able to bind to the ligand binding site and might therewith be less immune stimulating. The finding that β-(2,6)-fructans, as exopolysaccharide can activate TLR4, corroborates the findings of Xu *et al.*, who previously demonstrated that β-(2,6)-fructans isolated from *B. subtilis* exert activating effect on TLR4.^25^

Besides activation, also inhibition of TLR signalling was observed by β-(2,6)-fructans. Especially β-(2,6)-fructans exhibited strong inhibitory effects on TLR5. TLR5 is composed of a single-domain LRR structure that consists of an N-terminal β-hairpin capping motif and 13 complete LRR modules and two residues from LRR14.^47^ The concave surface is less regular than other TLRs and has a variation of helices and extended structures and specialized in recognizing flagellin. Little is known about how molecular structures such as mMW and hMW β-(2,6)-fructans are capable of inhibiting TLR5. Many pathogens, including flagellin-carrying organisms, use β-(2,6)-fructans as exopolysaccharides in their cell wall. It is, therefore, possible that the larger structures tested in this study bind to the ligand binding sites and are large enough to interfere with the activation of the receptor by the applied agonist flagellin of *S. typhimurium*.

Also, the endosomal TLR8 was inhibited by β-(2,6)-fructans. Although intracellularly located this finding might still have biological implications as the gastrointestinal tract is lined with cells that have phagocytic capacity. This includes intraepithelial dendritic cells as well as cells located in the Peyer patches such as M-cells, macrophages, and monocytes. Activation and inhibition of TLR8 activation is a multi-step process. For TLR8 activation the formation of an apo TLR8 dimer is needed and requires a proteolytic cleavage that induces a conformational change of TLR8 when the ligand is bound.^48^ For activation of TLR8 the ligand, *i.e.*, ssRNA, requires the formation of an activated dimer with the uridine part of ssRNA in the receptor.^49,50^ This is most likely where the β-(2,6)-fructans interferes with interaction with the receptors with van der Waals bonds and hydrogen bridges as the conformation and ability to bind in the agonist-activated TLR8 is different from that of the inactivated TLR8.^43,48,51^

Finally, we determined the combined outcome of activation of TLR2 and 4 and inhibition of TLR5 and 8 by incubating the different levans with dendritic cells. Also, TLR8 inhibition might be involved as phagocytosis of the fructans might occur in this set-up. However, only minor changes in cytokine levels were observed. Only for DCs directly stimulated with mMw fructans a significant reduction in MIP1α/CCL3 production was observed compared to the control. CCL3 is a member of the CC chemokine family and involved in the recruitment and activation of polymorphonuclear leukocytes after binding to receptors such as CCR1, CCR4, and CCR5. In addition, some trends towards enhanced MIP1α/CCL3, TNF-α and a molecular weight-dependent lowering of IL-10 were observed for the CSM-stimulated DCs, but there were no statistically significant differences compared to the untreated controls. This is most likely due to the counteracting effects of TLR2 and TLR4 stimulation and the inhibition of TLR5 and 8. Also, the addition of CSM to potentiate the responses of DCs to the stimuli^1,8^ did not significantly change the cytokine responses of the fructans.

Our study contributes to enhanced knowledge of the mechanisms that might be involved in immunomodulation induced by exopolysaccharides such as β-(2,6)-fructans. The molecules have a molecular weight-dependent effect on TLR signalling. The final effect on DC cytokine responses was a minor change in proinflammatory cytokines likely due to counteracting effects of activation and inhibition of TLRs. This however should not be interpreted as a suggestion that exopolysaccharides cannot be immune modulating. Exopolysaccharides are highly diverse in composition and different results might be expected from exopolysaccharides from other bacterial sources.^21^ Also, TLR expression is different under diseased and non-diseased conditions which might also impact the biological effects of exopolysaccharides such as of levans. Our study also demonstrates that fructans such as levans can impact TLR signalling just like has been shown for other fructans such as inulin-type and graminan-like fructans. It therewith opens new venues to use β-(2,6)-fructans as an immunomodulating component in disorders where TLR signalling is involved such as in allergies, mucositis and other intestinal disorders.^46,52–56^

## Author contributions

R. A., conceptualization; methodology; formal analysis; writing – original draft. M. M. P. O., conceptualization; methodology; investigation; writing – original draft. M. F., conceptualization; methodology; investigation; writing – original draft. C. F., investigation; writing – review & editing. B. J. H., investigation; writing – review & editing. M. M. F., investigation; writing – review & editing; supervision. M. T. C. W., conceptualization; resources; writing – original draft; supervision; funding acquisition. P. V., conceptualization; resources; writing – original draft; supervision; funding acquisition.

## Conflicts of interest

The authors declare no conflict of interest.

## Supplementary Material

FO-015-D3FO03066K-s001
