# Supplementary material for: Exopolysaccharide β-(2,6)-levan-type fructans have a molecular-weight-dependent modulatory effect on Toll-like receptor signalling
Source: Food Funct. 2023 Dec 18;15(2):676–88. doi: 10.1039/d3fo03066k (PMC10802977; doi:10.1039/d3fo03066k)
Supplement: FO-015-D3FO03066K-s001 [file FO-015-D3FO03066K-s001.pdf]

Electronic Supplementary Material (ESI) for Food & Function.
This journal is © The Royal Society of Chemistry 2023

Supplementary tables and figures

Exopolysaccharide β-(2,6)-levan-type fructans have a molecular-weight-dependent modulatory effect on Toll-Like Receptor signalling

Renate Akkerman [1*], Marjolein M. P. Oerlemans [1*], Michela Ferrari [2], Cynthia Fernández-Lainez [1,3,4], Bart J. de Haan [1], Marijke M. Faas [1], Marthe T. C. Walvoort [2] and Paul de Vos [1]

Supplementary table 1. Cell densities and positive controls used in reporter cell stimulation experiments

Cell line	Cell Density	Agonist	Agonist concentration
THP-1MD2-CD14	$1*10^6$ cells/mL	*E.coli* K12 lipopolysaccharide (LPS) Ultrapure	100 ng/mL
THP-1 DefMyD	$2*10^6$ cells/mL	L-Ala-gamma-D-Glu-mDAP (TriDAP)	10 µg/mL
HEK-Blue hTLR2	$2.8*10^5$ cells/mL	Heat-killed Listeria monocytogenes (HKLM)	10^7 cells/mL
HEK-Blue hTLR3	$2.8*10^5$ cells/mL	Poly(I:C) high molecular weight	1 mg/mL
HEK-Blue hTLR4	$1.4*10^5$ cells/mL	*E.coli* K12 lipopolysaccharide (LPS) Ultrapure	10 ng/mL
HEK-Blue hTLR5	$1.4*10^5$ cells/mL	Recombinant flagellin from *S. typhimurium* (Rec-FLA-ST)	100 ng/mL
HEK-Blue hTLR7	$2.2*10^5$ cells/mL	Imiquimod	5 µg/mL
HEK-Blue hTLR8	$2.2*10^5$ cells/mL	Single stranded RNA (ssRNA40/LyoVec TM)	5 µg/mL
HEK-Blue hTLR9	$4.5*10^5$ cells/mL	Type B CpG oligonucleotide (ODN 2006)	100 mg/mL

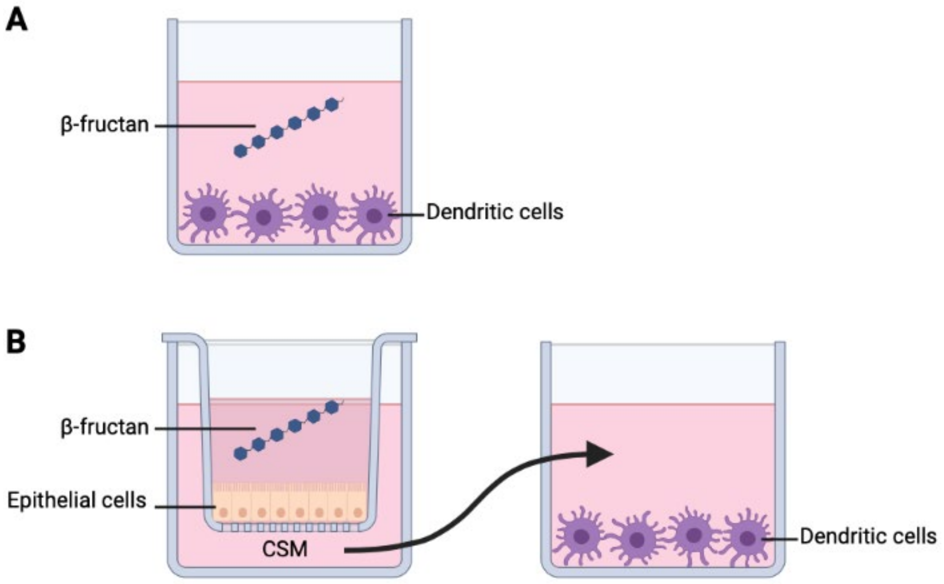

Supplementary figure 1: Design of the *in vitro* DC experiments. (A) DCs were incubated with β-fructans for 48 h to investigate their stimulatory properties and (B) to investigate the effect of soluble epithelial factors in response to incubation with β-fructans on DCs, Caco-2 cells were cultured on trans well inserts and incubated with β-fructans for 24 h on the apical side. Subsequently, Caco-spent medium (CSM) from the basolateral basin was incubated with DCs for another 48 h. DC supernatants were analyzed for cytokine production.

Supplementary table 2: Data under detection limit

Cytokine	Lower detection limit (µg/mL)	Interpretated value (µg/mL)
MCP-1/CCL2	-	-
MIP-1α/CCL3	13,4979	1,34979
IL-1β	2,97668	0,297668
IL-1RA	4645	464,5
IL-6	6,17284	0,617284
IL-10	5,28121	0,528121
TNFα	3,29218	0,329218

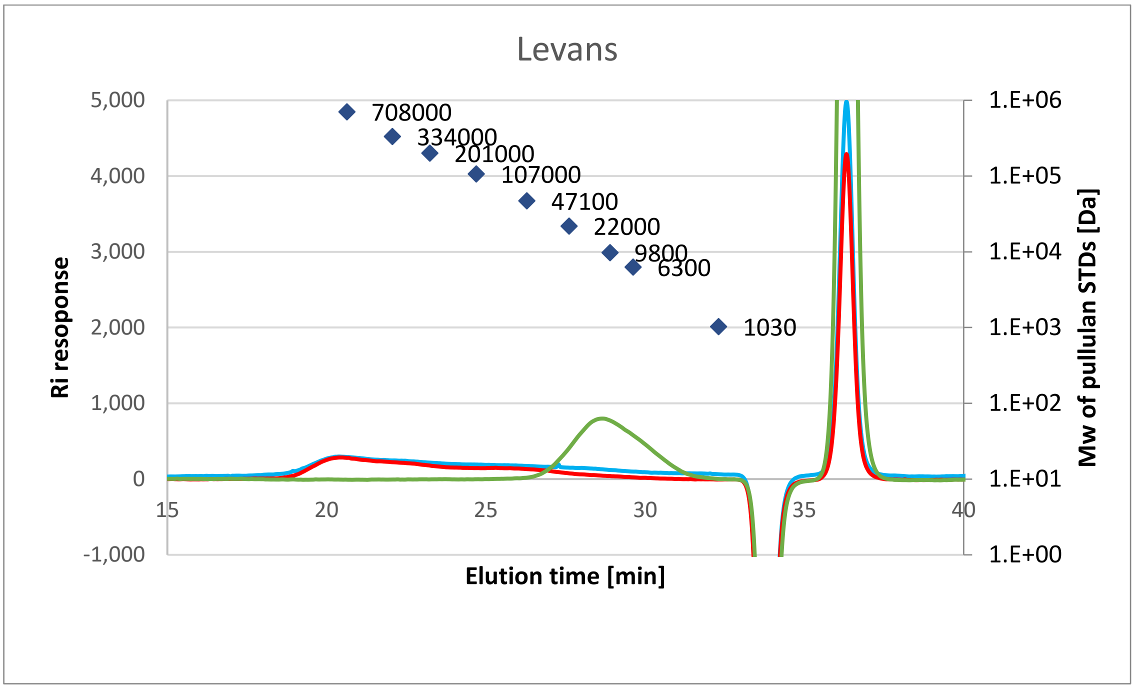

Supplementary figure 2: Molecular weight determination of the Levans included in this study measured by gel permeation chromatography (GPC). lMw β-(2,6)-fructan is represented in green. mMw β-(2,6)-fructan is represented in red and the hMw β-(2,6)-fructan is represented in blue. Blue dots on top of the plot represent the pullulan STDs.
